# Immunomodulatory factors in cervicovaginal secretions from pregnant and non-pregnant women: A cross-sectional study

**DOI:** 10.1186/1471-2334-11-263

**Published:** 2011-09-30

**Authors:** Jan Walter, Linda Fraga, Melanie J Orin, William D Decker, Theresa Gipps, Alice Stek, Grace M Aldrovandi

**Affiliations:** 1 Department of Plant Pathology and Microbiology, University of California, Riverside, USA; 2 LAC+USC Department of Obstetrics and Gynecology, Keck School of Medicine, University of Southern California, Los Angeles, USA; 3 Division of Infectious Diseases, Children's Hospital Los Angeles, University of Southern California, Los Angeles, USA

## Abstract

**Background:**

Pregnant women are at an increased risk for HIV infection due to unknown biological causes. Given the strong effect of sex-hormones on the expression of immunomuodulatory factors, the central role of mucosal immunity in HIV pathogenesis and the lack of previous studies, we here tested for differences in immunomuodulatory factors in cervico-vaginal secretions between pregnant and non-pregnant women.

**Methods:**

We compared concentrations of 39 immunomodulatory factors in cervicovaginal lavages (CVL) from 21 pregnant women to those of 24 non-pregnant healthy women from the US. We used Bonferroni correction to correct for multiple testing and linear regression modeling to adjust for possible confounding by plasma cytokine concentration, cervical ectopy, total protein concentration, and other possible confounders. Cervical ectopy was determined by planimetry. Concentration of immunomodulatory factors were measured by a multiplex assay, protein concentration by the Bradford Method.

**Results:**

Twenty six (66%) of the 39 measured immunomodulatory factors were detectable in at least half of the CVL samples included in the study. Pregnant women had threefold lower CVL concentration of CCL22 (geometric mean: 29.6 pg/ml versus 89.7 pg/ml, p = 0.0011) than non-pregnant women. CVL CCL22 concentration additionally correlated negatively with gestational age (Spearman correlation coefficient [R_S_]: -0.49, p = 0.0006). These associations remained significant when corrected for multiple testing.

CCL22 concentration in CVL was positively correlated with age and negatively correlated with time since last coitus and the size of cervical ectopy. However, none of these associations could explain the difference of CCL22 concentration between pregnant and non-pregnant women in this study, which remained significant in adjusted analysis.

**Conclusions:**

In this study population, pregnancy is associated with reduced concentrations of CCL22 in cervicovaginal secretions. The role of CCL22 on HIV transmission should now be investigated in prospective studies.

## Background

Pregnant women are at higher risk of acquiring Human Immunodeficiency Virus (HIV) infection compared to non-pregnant women [1-4]. If HIV positive, pregnant women may also transmit HIV more frequently to their uninfected partner than non-pregnant women (International Microbicides Conference 2010, abstract #8). The increased susceptibility for HIV infection during pregnancy is independent of sexual behavior and likely due to biological causes [1]. However, the underlying mechanisms for both the increased susceptibility and infectivity are unknown.

Previous studies have shown that sex hormones influence female genital tract immunity [5] and a large body of literature analyzed effects of proinflammatory cytokine concentration in cervicovaginal secretions with bacterial vaginosis and preterm birth [6]. However, comprehensive comparisons of cervicovaginal cytokine concentrations between pregnant and non-pregnant women have to our knowledge not been conducted.

Given the central role of cytokines and other mucosal immunomodulatory factors in HIV pathogenesis [7], and the profound systemic changes during immunity [8,9], we hypothesized that pregnancy may result in shifts of the cervicovaginal cytokine profile that may increase the risk of HIV infection. To explore this hypothesis, we conducted a comprehensive analysis of immunomuodulatory factors in samples collected from pregnant and non-pregnant women.

## Methods

### Study population

The study enrolled 23 pregnant and 25 non-pregnant women attending the Obstetrics and Gynecology clinic at the University of Southern California Medical Center in Los Angeles between February and April 2008. Healthy women between 17 and 45 years of age were invited to enroll in the study if they were: not on hormonal contraception in the last 6 months, had no intrauterine device, did not report sexual intercourse within the last 24 h and were not actively menstruating. All women underwent a clinical examination. Women with bacterial vaginosis or candidiasis were subsequently excluded from the analysis, resulting in a final study population of 21 pregnant and 24 non-pregnant women.

All women provided written consent and the study was approved by the institutional review board at the University of Southern California, Los Angeles, CA and Children's Hospital Los Angeles, CA.

### Data and sample collection

Socio-demographic, obstetric and gynecological data were collected by a structured questionnaire. A digital picture of the cervix was taken with an inserted endocervical wick (Tear-Flo™) serving as length standard. After removal of the endocervical wicks, a CVL sample was collected by bathing the cervical os in phosphate buffered saline (PBS). Fluid in the vaginal vault was then collected with a transfer pipette and stored on ice until transported to the laboratory for processing within 4 h. Blood was collected with ethylenediaminetetraacetic acid (EDTA) as anticoagulant and cells were separated from CVL sample and blood by low speed centrifugation. Supernatants were frozen at -80°C until measurement.

### Lab assays

Measurements of immunomodulatory factors were conducted with the Milliplex™ Map Human Cytokine/Chemokine KIT for the measurement of 39 premixed cytokines (Millipore, Billerica, MA) using the Luminex technology (Luminex Corporation, Austin, TX) as specified in manufacturer's instructions. Protein concentration in CVL was measured using the Quick-Start Bradford Dye Reagent (Bio-Rad, Hercules, CA) according to the manufacturer's instructions. All samples were assayed in duplicate and the mean between these measurements used for all analysis.

### Cervical ectopy

The size of the cervix as well as any visible endocervical epithelium covering the ectocervix was determined by planimetry and expressed in mm^2 ^using an image processing program (Adobe Photoshop)[10,11]. The degree of cervical ectopy was expressed as the percent of the visible endocervix covering the ectocervix.

### Statistical analysis

Concentrations outside of the range of the multiplex assay were imputed with a value just below the lower detection limit of the kit plus 2 standard deviations or above the upper cut-off (10,000 pg/ml) of the assay. To adjust for variation in the volume of phosphate buffered saline (PBS) used during the CVL (10 or 12 ml) concentrations determined from samples with lower dilution (10 ml) were divided by a factor of 1.2. Where appropriate, variables were log_10 _transformed to approximate normal distributions.

We used the chi-square test to compare categorical variables, unless the expected cell counts below 5, in which case we used Fisher's exact test. The chi-square trend test was used to compare ordered categorical variables, the Wilcoxon rank sum test for non-normally-distributed continuous variables, and the T-test for normally distributed variables. Bonferroni correction was used to correct for multiple testing. Pearson correlation coefficient were calculated for normally distributed and Spearman correlation coefficient for non-normally distributed variables.

Linear regression modeling was conducted to adjust for confounding. Variables were retained in the final model if they remained significantly associated with log_10_-transformed CCL22 concentration or if they changed the effect estimate by more than 15%. Two missing datapoints for the time since last coitus were imputed with the population median of 7 days to retain all women in the regression analysis. All statistical analyses were performed using SAS software (Version 9.2, Cary, NC).

## Results

### Characteristics of the study population

As expected, pregnant women more frequently reported vaginal discharge and had more pronounced cervical ectopy than non-pregnant women. Pregnant women were also younger than non-pregnant women. They, however, did not differ from non-pregnant women in other socio-economic or gynecological variables (Table 1).

**Table 1:** Characteristics of 21 pregnant and 24 non-pregnant women included in the analysis.

Variable	Pregnant women	Non-pregnant women	p-value
**Socio-demographic variables**			
Age [mean (STD)]	27.7 ± 6.0	33.8 ± 7.0	**0.003**
Hispanic [n (%)]	19 (90)	23 (96)	0.59
**Obstetric/gynecological variables**			
Gestational age [median weeks (IQR)]	28 (22-32)	/	
Parity [n (%)]:			
0 previous births	9 (43)	6 (26)	0.35
1-2 previous births	6 (29)	9 (39)	
3+ previous births	6 (29)	8 (35)	
Days since last coitus [median (IQR)]	7 (4, 15)	7 (3, 15)	0.63
Vaginal discharge [n (%)]	14 (67)	8 (33)	**0.03**
Vaginal bleeding during sample collection [n (%)]	9 (43)	5 (21)	0.11
Cervical ectopy [n (%)]	14 (67)	10 (42)	0.09
Size of ectopy among women with ectopy [median % (IQR)]	47 (32, 54)	17 (8, 26)	**0.008**
CVL protein concentration [median μg/ml (IQR)]	0.15 (0.07, 0.29)	0.10 (0.06, 0.18)	0.24

STD = standard deviation; IQR = interquartile range. Numbers may be slightly lower than the total due to missing data.

Twenty six (66%) of the 39 measured immunomodulatory factors were detectable in at least half of the CVL samples included in the analysis. With the exception of IL-1ra, none of immunomodulatory factors were frequently above the detection limit of the assay (Table 2).

**Table 2:** Concentrations of immunomodulatory factors in CVL of 21 pregnant and 24 non-pregnant women from Los Angeles.

	Total		Pregnant	Non-pregnant	p-value
Factor	n detectable*	Median (IQR) or geometric mean [pg/ml]^#^	Median (IQR) or geometric mean [pg/ml]^#^	Median (IQR) or geometric mean [pg/ml]^#^	
EGF	45	90.2 (75.1, 128.8)	89.0	102.6	0.33
Eotaxin	45	38.9 (28.5, 47.2)	36.1	40.7	0.43
FGF-2	34	11.1 (7.7, 21.5)	9.6 (< 3.2; 18.5)	8.9 (< 3.2; 17.8)	0.95
Flt-3 ligand	2	184.3 (14.9, 353.6)	< 4.6 (< 4.6; < 4.6)	< 4.6 (< 4.6; < 4.6)	0.97
Fractalkine	36	132.4(66.2, 293.6)	106.7(29.7; 171.7)	95.0 (11.2; 222.4)	0.92
G-CSF	45	383.4 (131.5, 741.6)	307.9	249.5	0.66
GM-CSF	36	77.8 (44.8, 137.3)	60.8 (18.8; 105.1)	64.6 (26.4; 123.5)	0.33
GRO	45	1,368.1 (756.0, 1,984.4)	1,175.8 (472.4; 2,328.0)	1,487.0 (1,109.0; 1,859.5)	0.49
IFNγ	42	2.0 (1.0, 3.8)	1.6	2.2	0.39
IFNα2	4	213.5 (93.1, 316.8)	< 40.6 (< 40.6; 40. < 6)	< 40.6 (< 40.6; < 40. 6)	0.98
IL-10	17	3.8 (1.1, 30.4)	< 0.5 (< 0.5; 1.3)	< 0.5 (< 0.5; 1.1)	0.90
IL-12p40	42	93.3 (58.2, 180.3)	105.7	86.2	0.48
IL-12p70	24	4.7 (2.5, 13.6)	0.9 (< 0.8; 3.5)	1.8 (< 0.8; 5.5)	0.49
IL-13	12	5.0 (1.9, 20.4)	< 0.9 (< 0.9; < 0.9)	< 0.9 (< 0.9; 1.7)	0.59
IL-15	18	4.0 (1.1, 5.6)	< 0.7 (< 0.7; 1.1)	< 0.7 (< 0.7; 4.0)	0.70
IL-17	45	3.0 (2.0, 4.2)	2.6	3.2	0.39
IL-1α	45	664.2 (278.9, 1,526.3)	812.8	502.5	0.15
IL-1β	28	30.4 (7.6, 49.8)	6.8 (< 0.7; 37.6)	6.9 (< 0.7; 40.5)	0.97
IL-1ra	45	> 10,000 (> 10,000, > 10,000)	> 10,000 (> 10,000, > 10,000)	> 10,000 (> 10,000, > 10,000)	0.37
IL-2	16	9.3 (1.6, 18.6)	< 0.6 (< 0.6; 6.6)	< 0.6 (< 0.6; 0.8)	0.15
IL-3	43	144.0 (69.8, 187.2)	155.5 (110.2; 187.2)	142.2 (58.5; 171.0)	0.65
IL-4	1	27.9	< 1.1 (< 1.1; < 1.1)	< 1.1 (< 1.1; < 1.1)	0.37
IL-5	12	0.1 (0.1, 0.3)	< 0.1 (< 0.1; < 0.1)	< 0.1 (< 0.1; 0.1)	0.51
IL-6	38	42.2 (22.1, 94.9)	34.1 (5.6; 91.7)	29.0 (9.1; 65.9)	1.00
IL-7	15	28.8 (7.6, 39.4)	< 4.0 (< 4.0; 17.1)	< 4.0 (< 4.0; 6.7)	0.74
IL-8	45	405.8 (254.3, 958.6)	526.0	429.4	0.52
IL-9	41	25.9 (6.8, 49.0)	11.6	18.4	0.28
IP-10	45	112.6 (40.5, 263.7)	97.7	144.2	0.29
MCP-1	44	79.3 (22.2, 208.6)	41.1	111.4	**0.03**
MCP-3	3	14.2 (6.0, 20.2)	< 3.7 (< 3.7; < 3.7)	< 3.7 (< 3.7; < 3.7)	0.62
CCL22	42	63.2 (25.9, 131.9)	29.7	89.7	**0.001**
MIP-1α	44	62.0 (36.6, 86.0)	54.3	57.5	0.79
MIP-1β	35	55.2 (30.9, 108.9)	35.3 (9.5; 69.9)	46.8 (22.0; 91.5)	0.52
sCD40L	8	52.0 (24.5, 98.8)	< 9.0 (< 9.0; < 9.0)	< 9.0 (< 9.0; < 9.0)	0.90
sIL-2Ra	6	22.9 (15.0, 27.5)	< 7.7 (< 7.7; < 7.7)	< 7.7 (< 7.7; < 7.7)	0.60
TGFα	38	14.4 (5.9, 24.0)	9.5 (2.8; 26.5)	12.9 (3.8; 17.3)	0.72
TNFα	43	0.8 (0.5, 2.8)	0.8 (0.5; 2.4)	0.9 (0.5; 2.5)	0.84
TNFβ	13	7.1 (5.0, 13.6)	< 3.4 (< 3.4; 4.3)	< 3.4 (< 3.4; 4.7)	1.00
VEGF	43	56.2 (37.1, 102.6)	53.3	73.9	0.29

IQR = interquartile range

* number of samples with concentrations above the detection limit of the kit plus 2 standard deviations as provided by the manufacturer;

# undetectable samples were imputed with a values just below the cut off, geometric means (a single number) are shown for variables normally distributed on the log scale, median and interquartile range are shown for non-normally distributed variables

### Pregnancy and immunomodulatory factors in CVL

CVL collected from pregnant women contained threefold lower concentrations of C-C motif chemokine 22 (CCL22; also known as macrophage-derived chemokine) than CVL from non-pregnant women (mean ± standard deviation [SD] of log_10 _pg CCL22 per ml 1.5 ± 0.4 versus 2.0 ± 0.5, p = 0.0011, geometric mean: 29.7 pg/ml vs 89.7 pg/ml). Pregnant women also had lower CVL concentrations of monocyte chemotactic protein-1 (MCP-1) than non-pregnant women (mean ± SD of log_10 _pg per ml among pregnant women: 1.6 ± 0.6 versus non-pregnant women 2.0 ± 0.7, p = 0.03; geometric mean: 41.1 pg/ml versus 111.4 pg/ml); however, only the difference in CCL22 remained significant when adjusted for multiple testing (p < 0.0013).

**Figure 1:**
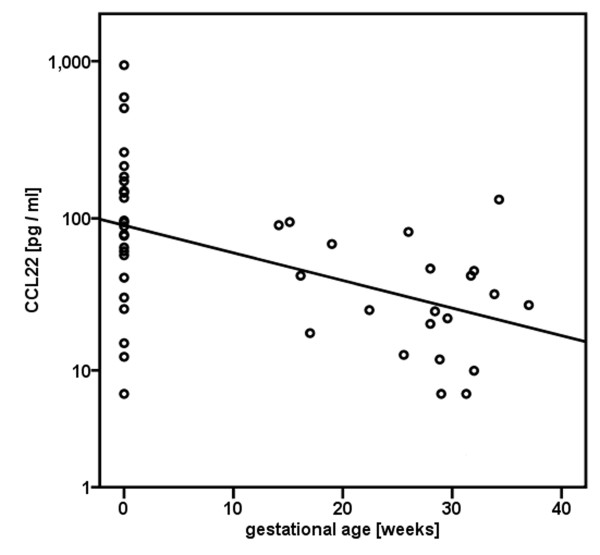
**Scatter plot of CCL22 concentration in cervicovaginal lavages with gestational age among 21 pregnant and 24 non-pregnant women**. Non-pregnant women were assigned a gestational age of 0 weeks.

The difference in CVL CCL22 concentration between pregnant and non-pregnant women strengthened slightly when pregnant women were restricted to those in their third trimester (n = 13) (mean ± standard deviation [SD] of log_10 _pg CCL22 per ml 1.4 ± 0.4 versus 2.0 ± 0.5, p = 0.0011, geometric mean: 23.6 pg/ml vs 89.7 pg/ml). There additionally was a strong negative correlation between gestational age and CCL22 concentration in CVL (Spearman correlation coefficient [R_S_]: -0.49, p = 0.0006) when analyzed in the whole population assigning a gestational age of "0" to non-pregnant women (Figure 1). There was, however, no significant association of CCL22 concentration with gestational age when restricted to pregnant women (R_S _= -0.21, p = 0.34)

### Correlation of CVL CCL22 concentration with other immunomodulatory factors

As listed in Table 3, the concentration of CCLL22 concentration correlated or tended to correlated with that of Eotaxin, Fractalkine, GM-CSF, GRO, IL-17, IL-9, IP-10, MCP-1, MCP-3, TGFα, TNFβ and VEGF in CVL. There was, however, no correlation with the total protein concentration in CVL (R_S _= 0.10; p = 0.51) or with plasma CCL22 concentration (R_S _= 0.06, p = 0.69).

**Table 3:** Immunomodulatory factors associated with CCL22 in CVL

Factor	Correlation Coefficient	p-value
Eotaxin	R_P _= 0.38	0.01
Fractalkine	R_S _= 0.26	0.09
GM-CSF	R_S _= 0.43	0.003
GRO	R_S _= 0.38	0.01
IL-17	R_P _= 0.33	0.03
IL-9	R_P _= 0.28	0.06
IP-10	R_P _= 0.61	< .0001
MCP-1	R_P _= 0.57	< .0001
MCP-3	R_S _= 0.25	0.10
TGFα	R_S _= 0.29	0.05
TNFβ	R_S _= 0.26	0.08
VEGF	R_P _= 0.41	0.005

All concentrations were in log_10 _pg/ml. R_P _= Pearson correlation coefficient; R_S _= Spearman correlation coefficient

### Can the difference in CVL CCL22 concentration between pregnant and non-pregnant women be explained by other factors?

To test whether underlying differences between pregnant and non-pregnant women may have caused the difference in CCL22 concentration, we conducted linear regression modeling adjusting for possible confounders. In univariate analysis, age, time since last coitus and the size of cervical ectopy were additionally associated with CCL22 concentration in CVL (Table 4). When adjusted for age and time since last coitus, the association between pregnancy and CCL22 concentrations remained strong. None of the other variables shown in Table 1, including cervical ectopy or vaginal discharge, remained significantly associated with the CCL22 concentration or appreciably changed the effect estimate when additionally included in the model. The results are similar, when gestational age instead of pregnancy is included in the model or when the CCL22 concentration is expressed as ratio of CCL22 to total protein in CVL to adjust for possible variation during the sample collection.

**Table 4:** Linear regression modeling of log_10 _transformed CCL22 concentrations in CVL among 21 pregnant and 24 non-pregnant women.

Variable	Unadjusted regression coefficient (95%-CI)	Adjusted regression coefficient (95%-CI)*
Pregnancy	-0.48 (-0.76, -0.20)	-0.38 (-0.66, -0.10)
Age per year increase	0.02 (0.003, 0.05)	0.02 (-0.004, 0.04)
Ecotopy per % increase	-0.007 (-0.01, -0.0002)	/
Time since last coitus per log_10 _day increase	-0.30 (-0.54, -0.06)	-0.32 (-0.53, -0.11)

CI = confidence interval

*adjusted for all variables shown

## Discussion

Among this group of healthy American women, we found threefold lower concentration of CCL22 in CVL samples from pregnant women than in those from non-pregnant women. This difference remained significant when corrected for multiple testing or in adjusted analysis, suggesting that pregnancy may result in reduced concentration of cervicovaginal CCL22.

The strength of this study lies in the large number of analyzed cytokines and immunomodulatory factors among healthy women. To our knowledge, this is the most comprehensive analysis of these factors in CVL of pregnant and non-pregnant women. In contrast to at least some previous studies [12-15], we did not detect differences in proinflammatory cytokines between pregnant and non-pregnant women. This could be explained by the sample size, the exclusion of women with clinical bacterial vaginosis, the relatively early collection of samples or fluctuations of these cytokines throughout pregnancy. We are however the first study that tested CCL22 concentrations in CVL.

CCL22 has previously been detected in other mucosal sites including the intestine [16], the lung [17] and the endometrium [18] as well as in vaginal tissue in mice [19], thus its presence in vaginal tissues among humans seems plausible.

Previous studies have described fluctuations of CCL22 expression in endometrium during the menstrual cycle and increases in the same tissue during early pregnancy [20], suggesting a control by sex hormones. While it is unclear, what caused the decreased CCL22 concentrations among pregnant women in our study, progesterone has been shown to suppress the NF-κB transcription factor [21], which is an activator of CCL22 expression [22]. It is therefore possible that increased progesterone concentrations directly result in reduced CCL22 expression, which should be tested in vitro. In addition, CCL22 in our analysis was associated with a number of immunomodulatory factors, especially Ip10 and MCP-1. It was also increased shortly after coitus. Thus it is likely that there are a number of other physiological and immunological mechanisms that also influence CCL22 concentrations in CVL [23].

Intriguingly, CCL22 has been implied in the HIV pathogenesis in several ways. CCL22 is a T-helper cell type (TH) 2 cytokine that is highly expressed in macrophages and dendritic cells of the monocyte line [17] as well as in activated T-cells [24]. It is a strong chemoattractant for leukocytes expressing the CCR4 receptor [23] and has been suggested to be a key regulator of innate immunity in mice [25]. In at least some in vitro studies, CCL22 has been suggested to have HIV suppressive effects [26-29]. Such mechanisms could explain the increased risk of HIV infection with decreased CCL22 concentration. However, at least one other study suggested that CCL22 is secreted by CD16+ monocyte-derived macrophages to activate resting T-cells for HIV infection [30] and may therefore also increase the risk of HIV infection in certain situation. Thus further analysis of the effects of CCL22 on mucosal cytokine concentration is required.

As in all statistical analysis, we cannot exclude that differences in CVL concentrations of CCL22 are caused by chance. However, given the strong difference observed here, its possible regulation by sex hormones and its possible implication in HIV pathogens, a role of CCL22 in mediating a protection against HIV at the female genital mucosa seems plausible and should be investigated further.

## Conclusion

In this cohort, pregnancy is associated with reduced CCL22 concentration in cervicovaginal secretion, which may influence the risk of HIV infection.

## List of abbreviations

HIV: human immunodeficiency virus; CVL: cervicovaginal lavage; EDTA: ethylenediaminetetraacetic acid; STD: standard deviation; IQR: interquartile range; R_P _: Pearson correlation coefficient; R_S _: Spearman correlation coefficient; PBS: phosphate buffered saline; CCL22: C-C motif chemokine 22; MCP-1: monocyte chemotactic protein-1; GM-CSF: granulocyte macrophage colony-stimulating factor; GRO: growth regulated oncogene; MCP-3: monocyte chemotactic protein-3; TGFα: transforming growth factor alpha; TNFβ: tumor necrosis factor beta; VEGF: vascular endothelial growth factor.

## Competing interests

The authors declare that they have no competing interests.

## Authors' contributions

GMA and AS designed the study. LF, AS and TG collected the samples and clinical data. WDD and JW oversaw the laboratory. MJO conduced part of the laboratory work and helped with the analysis. JW conducted the analysis and wrote the manuscript. All authors have reviewed and approved the manuscript.

## Pre-publication history

The pre-publication history for this paper can be accessed here:

http://www.biomedcentral.com/1471-2334/11/263/prepub

## References

[B1] Gray RH Li X Kigozi G Serwadda D Brahmbhatt H Wabwire-Mangen F Nalugoda F Kiddugavu M Sewankambo N Quinn TC Increased risk of incident HIV during pregnancy in Rakai, Uganda: a prospective study Lancet 2005 366 9492 1182 1188 10.1016/S0140-6736(05)67481-8 16198767

[B2] Leroy V Van de Perre P Lepage P Saba J Nsengumuremyi F Simonon A Karita E Msellati P Salamon R Dabis F Seroincidence of HIV-1 infection in African women of reproductive age: a prospective cohort study in Kigali, Rwanda, 1988-1992 Aids 1994 8 7 983 986 10.1097/00002030-199407000-00017 7946110

[B3] Taha TE Dallabetta GA Hoover DR Chiphangwi JD Mtimavalye LA Liomba GN Kumwenda NI Miotti PG Trends of HIV-1 and sexually transmitted diseases among pregnant and postpartum women in urban Malawi Aids 1998 12 2 197 203 10.1097/00002030-199802000-00010 9468369

[B4] Gray RH Wabwire-Mangen F Kigozi G Sewankambo NK Serwadda D Moulton LH Quinn TC O'Brien KL Meehan M Abramowsky C Randomized trial of presumptive sexually transmitted disease therapy during pregnancy in Rakai, Uganda Am J Obstet Gynecol 2001 185 5 1209 1217 10.1067/mob.2001.118158 11717659

[B5] Wira CR Fahey JV Ghosh M Patel MV Hickey DK Ochiel DO Sex hormone regulation of innate immunity in the female reproductive tract: the role of epithelial cells in balancing reproductive potential with protection against sexually transmitted pathogens Am J Reprod Immunol 2010 63 6 544 565 10.1111/j.1600-0897.2010.00842.x 20367623 PMC3837356

[B6] Krupa FG Faltin D Cecatti JG Surita FG Souza JP Predictors of preterm birth Int J Gynaecol Obstet 2006 94 1 5 11 10.1016/j.ijgo.2006.03.022 16730012

[B7] Iqbal SM Kaul R Mucosal innate immunity as a determinant of HIV susceptibility Am J Reprod Immunol 2008 59 1 44 54 18154595 10.1111/j.1600-0897.2007.00563.x

[B8] Szarka A Rigo J Jr Lazar L Beko G Molvarec A Circulating cytokines, chemokines and adhesion molecules in normal pregnancy and preeclampsia determined by multiplex suspension array BMC Immunol 2010 11 59 10.1186/1471-2172-11-59 21126355 PMC3014878

[B9] Toldi G Rigo J Jr Stenczer B Vasarhelyi B Molvarec A Increased prevalence of IL-17-producing peripheral blood lymphocytes in pre-eclampsia Am J Reprod Immunol 2011 66 3 223 229 10.1111/j.1600-0897.2011.00987.x 21306467

[B10] Morrison CS Bright P Blumenthal PD Yacobson I Kwok C Zdenek S Pan Z Computerized planimetry versus clinical assessment for the measurement of cervical ectopia Am J Obstet Gynecol 2001 184 6 1170 1176 10.1067/mob.2001.113125 11349184

[B11] Jacobson DL Peralta L Farmer M Graham NM Gaydos C Zenilman J Relationship of hormonal contraception and cervical ectopy as measured by computerized planimetry to chlamydial infection in adolescents Sex Transm Dis 2000 27 6 313 319 10.1097/00007435-200007000-00003 10907905

[B12] Beigi RH Yudin MH Cosentino L Meyn LA Hillier SL Cytokines, pregnancy, and bacterial vaginosis: comparison of levels of cervical cytokines in pregnant and nonpregnant women with bacterial vaginosis J Infect Dis 2007 196 9 1355 1360 10.1086/521628 17922400

[B13] Donders GG Vereecken A Bosmans E Spitz B Vaginal cytokines in normal pregnancy Am J Obstet Gynecol 2003 189 5 1433 1438 10.1067/S0002-9378(03)00653-7 14634582

[B14] Luo L Ibaragi T Maeda M Nozawa M Kasahara T Sakai M Sasaki Y Tanebe K Saito S Interleukin-8 levels and granulocyte counts in cervical mucus during pregnancy Am J Reprod Immunol 2000 43 2 78 84 10.1111/j.8755-8920.2000.430203.x 10735598

[B15] Sennstrom MB Ekman G Westergren-Thorsson G Malmstrom A Bystrom B Endresen U Mlambo N Norman M Stabi B Brauner A Human cervical ripening, an inflammatory process mediated by cytokines Mol Hum Reprod 2000 6 4 375 381 10.1093/molehr/6.4.375 10729321

[B16] Berin MC Dwinell MB Eckmann L Kagnoff MF Production of MDC/CCL22 by human intestinal epithelial cells Am J Physiol Gastrointest Liver Physiol 2001 280 6 G1217 1226 11352815 10.1152/ajpgi.2001.280.6.G1217

[B17] Godiska R Chantry D Raport CJ Sozzani S Allavena P Leviten D Mantovani A Gray PW Human macrophage-derived chemokine (MDC), a novel chemoattractant for monocytes, monocyte-derived dendritic cells, and natural killer cells J Exp Med 1997 185 9 1595 1604 10.1084/jem.185.9.1595 9151897 PMC2196293

[B18] Jones RL Hannan NJ Kaitu'u TJ Zhang J Salamonsen LA Identification of chemokines important for leukocyte recruitment to the human endometrium at the times of embryo implantation and menstruation J Clin Endocrinol Metab 2004 89 12 6155 6167 10.1210/jc.2004-0507 15579772

[B19] Lindqvist M Navabi N Jansson M Samuelson E Sjoling A Orndal C Harandi AM Local cytokine and inflammatory responses to candidate vaginal adjuvants in mice Vaccine 2009 28 1 270 278 10.1016/j.vaccine.2009.09.083 19800444

[B20] Jones RL Morison NB Hannan NJ Critchley HO Salamonsen LA Chemokine expression is dysregulated in the endometrium of women using progestin-only contraceptives and correlates to elevated recruitment of distinct leukocyte populations Hum Reprod 2005 20 10 2724 2735 10.1093/humrep/dei140 15979999

[B21] Kelly RW King AE Critchley HO Cytokine control in human endometrium Reproduction 2001 121 1 3 19 10.1530/rep.0.1210003 11226025

[B22] Poole E Atkins E Nakayama T Yoshie O Groves I Alcami A Sinclair J NF-kappaB-mediated activation of the chemokine CCL22 by the product of the human cytomegalovirus gene UL144 escapes regulation by viral IE86 J Virol 2008 82 9 4250 4256 10.1128/JVI.02156-07 18287226 PMC2293074

[B23] Mantovani A Gray PA Van Damme J Sozzani S Macrophage-derived chemokine (MDC) J Leukoc Biol 2000 68 3 400 404 10985257

[B24] Romano JW Shurtliff RN Grace M Lee EM Ginocchio C Kaplan M Pal R Macrophage-derived chemokine gene expression in human and macaque cells: mRNA quantification using NASBA technology Cytokine 2001 13 6 325 333 10.1006/cyto.2001.0843 11292315

[B25] Matsukawa A Hogaboam CM Lukacs NW Lincoln PM Evanoff HL Kunkel SL Pivotal role of the CC chemokine, macrophage-derived chemokine, in the innate immune response J Immunol 2000 164 10 5362 5368 10799899 10.4049/jimmunol.164.10.5362

[B26] Abdelwahab SF Cocchi F Bagley KC Kamin-Lewis R Gallo RC DeVico A Lewis GK HIV-1-suppressive factors are secreted by CD4+ T cells during primary immune responses Proc Natl Acad Sci USA 2003 100 25 15006 15010 10.1073/pnas.2035075100 14657379 PMC299882

[B27] Pal R Garzino-Demo A Markham PD Burns J Brown M Gallo RC DeVico AL Inhibition of HIV-1 infection by the beta-chemokine MDC Science 1997 278 5338 695 698 10.1126/science.278.5338.695 9381181

[B28] Cota M Mengozzi M Vicenzi E Panina-Bordignon P Sinigaglia F Transidico P Sozzani S Mantovani A Poli G Selective inhibition of HIV replication in primary macrophages but not T lymphocytes by macrophage-derived chemokine Proc Natl Acad Sci USA 2000 97 16 9162 9167 10.1073/pnas.160359197 10908681 PMC16839

[B29] Agrawal L Vanhorn-Ali Z Alkhatib G Multiple determinants are involved in HIV coreceptor use as demonstrated by CCR4/CCL22 interaction in peripheral blood mononuclear cells (PBMCs) J Leukoc Biol 2002 72 5 1063 1074 12429730

[B30] Ancuta P Autissier P Wurcel A Zaman T Stone D Gabuzda D CD16+ monocyte-derived macrophages activate resting T cells for HIV infection by producing CCR3 and CCR4 ligands J Immunol 2006 176 10 5760 5771 16670281 10.4049/jimmunol.176.10.5760

